# Prognostic value of serum levels of interleukin 6 and of serum and plasma levels of vascular endothelial growth factor in hormone-refractory metastatic breast cancer patients

**DOI:** 10.1038/sj.bjc.6600956

**Published:** 2003-05-27

**Authors:** T Bachelot, I Ray-Coquard, C Menetrier-Caux, M Rastkha, A Duc, J-Y Blay

**Affiliations:** 1 Unité Cytokine et Cancer, INSERM U-453 and Centre Léon Bérard, 28 rue Laënnec 69008 Lyon, France

**Keywords:** multivariate analysis, survival rate, human, biological markers

## Abstract

Prediction of survival for patients with metastatic breast cancer is often inaccurate and may be helped by new biological parameters. Tumour growth being angiogenesis-dependent, it has been hypothesised that the assessment of angiogenic factor production might reflect the clinical behaviour of cancer progression. This study was designed to investigate the clinical significance of vascular endothelial growth factor (VEGF) and interleukin 6 (IL-6) in hormone-refractory metastatic breast cancer. Serum and plasma concentrations of VEGF and serum concentration of IL-6 were measured in 87 patients with a fully documented history of metastatic breast cancer using an enzyme-linked immunoassay. All patients had detectable levels of VEGF, whereas 39% patients had detectable serum levels of IL-6. There was a positive correlation between IL-6 levels and the theoretical VEGF load of platelets (*P*<0.001). The presence of high levels of serum IL-6, but not VEGF, was significantly correlated to a shorter survival. In a multivariate analysis along with clinical prognostic parameters, serum IL-6 was identified as an independent adverse prognostic variable for overall survival (*P*<0.001). These results indicate that serum IL-6 levels correlate to poor survival in patients with hormone-refractory metastatic breast cancer. Vascular endothelial growth factor serum and plasma levels are not useful indicators of prognosis for these patients.

Although several prognostic factors for survival have been identified in metastatic breast cancer patients, the prognosis of individual patients frequently fails to confirm published predictions (Honig, 1996). Additional biological parameters are therefore required to gain more accurate insight into the outcome of metastatic breast cancer patients. Recent cancer research has highlighted the importance of tumour neovascularisation in the initial and metastatic growth of cancer (Hanahan and Folkman, 1996) and there is solid evidence supporting the evaluation of tumour angiogenesis for prognosis in cancer patients (Poon *et al*, 2001). Thus, the assessment of angiogenic factor production may allow a better estimation of survival for patients with metastatic breast cancer.

The vascular endothelial growth factor (VEGF) is one of the most potent and specific angiogenic molecules. In breast cancer, VEGF expression in the primary tumour appears to be significantly correlated with relapse-free survival (Gasparini, 2000). Elevated serum levels of VEGF have been found in metastatic cancer patients. These levels have been correlated to the clinical course of the disease in several tumour types (Poon *et al*, 2001; Ugurel *et al*, 2001; Molica *et al*, 2002). As most serum VEGF is released from platelets during blood clotting, it has been suggested that plasma VEGF may be a better reflection of ongoing angiogenic activity, although this assumption has been challenged (Banks *et al*, 1998; Salven *et al*, 1999a; Wynendaele *et al*, 1999; George *et al*, 2000). To date, plasma VEGF has not been shown to be a better prognostic factor than serum VEGF in any solid tumour (Werther *et al*, 2002).

Interleukin 6 (IL-6) is a pleiotropic cytokine produced by a variety of cell types, including endothelial cells and normal haematopoietic cells (Kishimoto *et al*, 1995). High IL-6 serum levels are correlated with shorter survival in patients with haematological malignancies, renal cell carcinoma and prostate cancer (Blay *et al*, 1992; Nakashima *et al*, 2000). Recent publications have highlighted the importance of IL-6 in the regulation of VEGF production. Interleukin 6 expression is induced by hypoxia and, in turn, is able to upregulate VEGF transcription via a specific response element present upstream of the transcription initiation site of VEGF (Cohen *et al*, 1996; Yan *et al*, 1997). Furthermore, IL-6 has been shown to correlate with platelet count and platelet VEGF content. As circulating VEGF is mostly transported by platelets, IL-6 could be regarded as an indirect angiogenic factor that facilitates the production and the distribution of VEGF to metastatic sites (Salgado *et al*, 1999,^2002^; Vermeulen *et al*, 1999).

The present study was undertaken to investigate connections between serum VEGF, plasma VEGF and serum IL-6 levels in metastatic breast cancer patients and evaluate their potential relation to the clinical outcome.

## PATIENTS AND METHODS

### Patients

Serum and matched plasma samples from 73 patients were used in this study. A serum sample without matched plasma sample was available for an additional 14 patients. All patients had a fully documented history of metastatic breast cancer and were treated in our institution between January 1995 and November 2000. Serums and EDTA plasma samples were collected after obtaining informed consent, aliquoted and stored at −80°C. The characteristics of the patients are shown in Table 1

**Table 1:** Patient characteristics

**Characteristics**	**No. of patients**	**Percent**
Entered on study	87	100
*Age (years)*		
Median	54	
Range	26–86	
*PS*		
0–1	55	63
2–4	32	37
*Receptor status*		
ER Positive	58	67
ER Negative	23	26
Unknown	6	7
*Adjuvant chemotherapy*		
Received	41	47
Not received	46	53
*Prior chemotherapy for metastatic disease*		
Received	61	70
Not received	26	30
*Disease-free interval*		
<24 months	49	56
>24 months	38	44
*Metastatic site*		
Liver	51	59
Lung	45	48
One or two	49	56
Three or more	38	44
*LDH*		
⩽1 × normal	25	29
>1 × normal	62	71

PS=performance status; ER=estrogen receptor; LDH=lactate dehydrogenase.

. Median age was 54 years (range 26–86). In all, 41 patients (47%) had received adjuvant chemotherapy as part of initial therapy. The median disease-free interval (from first diagnosis to recurrence) of the 87 patients was 32 months (range 0–19 years). For the 41 patients who had received adjuvant chemotherapy, there was at least a 4-month interval between the last course of adjuvant chemotherapy and blood sampling. All patients in this series had metastatic disease refractory to hormone therapy, that is, receptor-negative for both oestrogen and progesterone or progressive after hormone therapy. In all, 61 patients had previously received first-line chemotherapy for metastatic disease. Most patients (75%) had metastatic disease at multiple sites and 59% had liver involvement.

### Serum analysis

Serum and EDTA plasma samples were kept frozen at −80°C, then thawed shortly before determination of VEGF and IL-6 levels. Commercially available immunoassay kits were used according to the manufacturers' instructions. Vascular endothelial growth factor levels were determined with the Quantikine® human VEGF immunoassay kit from R&D system (R&D System, Minneapolis, MN, USA); IL-6 levels were determined with the Human IL-6 Enzyme Immunoassay kit from Immunotech (Immunotech, Marseille, France). Both assays employ the quantitative sandwich enzyme immunoassay technique. All plasma and serum level determinations were performed in triplicate. The theoretical detection limits of the immunoassay were 5 pg ml^−1^ for VEGF and 8 pg ml^−1^ for IL-6.

### Statistical analysis

Statistical analyses were carried out according to the procedures of the SPSS® package 8.0 (Chicago, USA, 1999). Patients' characteristics were compared using Student's *t*-test for quantitative variables. Correlation analysis with the Pearson test was used to study relations between continuous variables. Survival data, considered from the day of sampling to the time of death or the time of last follow-up, were assessed by means of the Kaplan–Meier method. Univariate analyses of survival times were performed using the log rank test. For this analysis, we tested three different threshold values for each of the two variables: the lowest quartile, the median and the highest quartile. In addition, we tested the detection limit for IL-6 and two threshold values previously reported as relevant for VEGF (Salven *et al*, 2000; Ugurel *et al*, 2001). The Cox proportional hazard regression model was used to identify independent prognostic factors for survival. The variables introduced in the model were prognostic factors commonly accepted for metastatic breast cancer and parameters previously identified as significantly related to survival in the univariate analysis.

## RESULTS

### Serum and plasma levels of VEGF and serum levels of IL-6

Serum VEGF was detectable in all 87 patients. The median serum VEGF level was 302 pg ml^−1^ (range 31–3183 pg ml^−1^). Confirming previously published results (Salgado *et al*, 1999), high serum VEGF levels were correlated with high platelet counts in our patients (*r*=0.6; *P*<0.001). As previously described, we estimated the theoretical VEGF load of platelets as the ratio of VEGF serum concentration on platelet number (Banks *et al*, 1998; Salgado *et al*, 1999; Salven *et al*, 1999a). As calculated, the median platelet VEGF concentration was 1.5 pg 10^−6^ platelets (range 0.2–9.6 pg 10^−6^ platelets).

**Figure 1:**
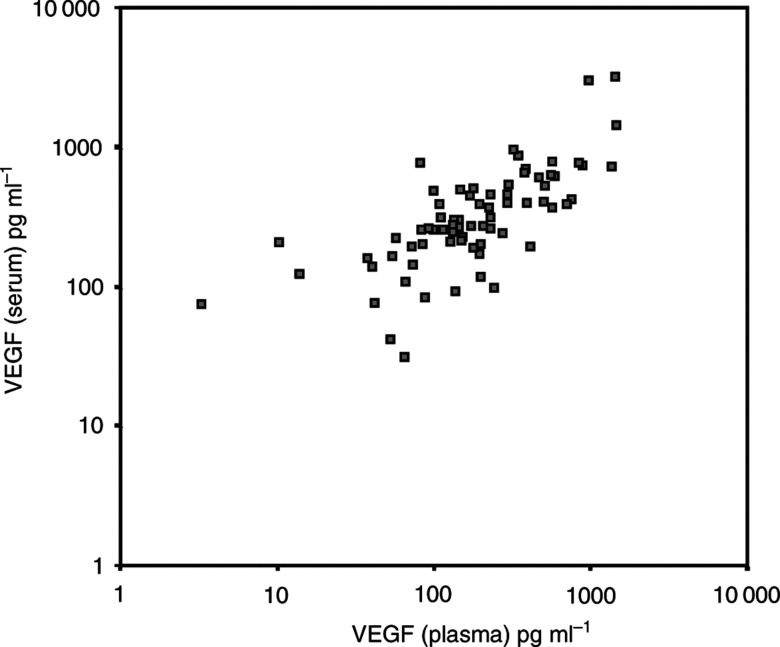
Correlation of plasma VEGF and serum VEGF in metastatic breast cancer patients (*r*=0.74, *P*<0.001).

Plasma VEGF levels were evaluated in matched plasma samples available for 73 patients. The median plasma VEGF level was 177 pg ml^−1^ (range 0–1447 pg ml^−1^). Plasma VEGF was not detectable in one patient (1.3%). There was a strong correlation between serum and plasma VEGF concentrations (*r*=0.74, *P*<0.001, Figure 1

), and between plasma VEGF and platelet count (*r*=0.5; *P*<0.001).

Interleukin-6 serum levels, assessed on 80 patients, were detectable in 31 (39%), with a median of 30 pg ml^−1^ when detectable (range 8.2–211 pg ml^−1^). In the following analysis, IL-6 serum level is considered to be 0 pg ml^−1^ for the 49 patients whose serum level was below the detection limit (8 pg ml^−1^). The correlation between IL-6 levels and platelet counts was not statistically significant (*r*=−0.08, *P*=0.5), neither was the correlation between serum IL-6 and serum VEGF levels (*r*=0.16, *P*=0.15) or plasma VEGF levels (*r*=0.07, *P*=0.5). On the other hand, we found a positive correlation between serum IL-6 levels and the theoretical VEGF load of platelets (*r*=0.54, *P*<0.001). The mean VEGF load of platelets was 2.8 pg 10^−6^ platelets for the 15 patients whose serum IL-6 level was above 30 pg ml^−1^, *vs* 1.7 pg 10^−6^ platelets for the 65 patients whose serum IL-6 level was below 30 pg ml^−1^ (*P*=0.01). With regard to disease extension, we did not find any correlation between IL-6 levels and liver involvement or IL-6 levels and the number of metastatic sites.

### Prognostic value of IL-6 and VEGF for overall survival

At the time of analysis, the median survival of the entire patient population was 10 months from the date of sampling. The actuarial survival rates were 44 and 25% at 12 and 24 months, respectively.

**Figure 2:**
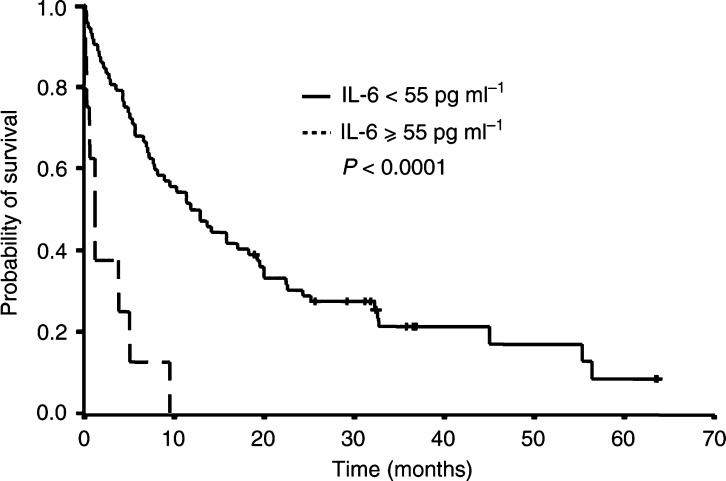
Survival of patients as a function of serum IL-6 levels. Patients with high levels of serum IL-6 constituted a subgroup of very poor prognosis. The cutoff value (55 pg ml^−1^) represents the highest quartile for serum IL-6 levels when detectable.

Serum IL-6 levels correlated with survival. Survival differences were statistically significant for three of the four cutoff values tested in univariate analysis: detection limit (8 pg ml^−1^, *P*=0.07), lowest quartile (13 pg ml^−1^, *P*=0.02), median (30 pg ml^−1^, *P*=0.05) and highest quartile (55 pg ml^−1^, *P*<0.001). High serum IL-6 levels were associated with a particularly poor survival: A serum IL-6 level over 13 pg ml^−1^ was associated with a median survival time of 4 months, whereas patients with serum IL-6 level below 13 pg ml^−1^ had a median survival time of 13 months. The median survival time of the eight patients with serum IL-6 levels above the highest quartile (55 pg ml^−1^) was 1 month, *vs* 12 months for the 72 patients with IL-6 levels below 55 pg ml^−1^ (Figure 2

).

**Figure 3:**
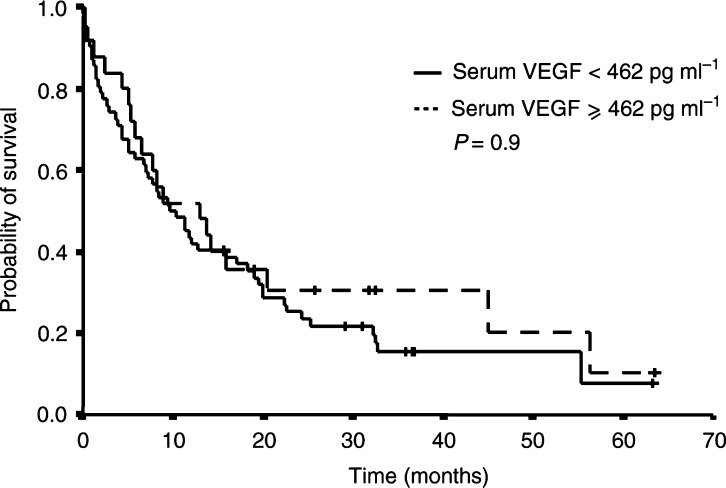
Survival of patients as a function of serum VEGF levels. We could not determine any threshold value that would make it possible to discriminate patients into two subgroups of different prognosis.

In contrast, no correlation between survival and serum or plasma VEGF levels was observed. Different threshold values of serum VEGF levels have been tested, particularly 363 and 462 pg ml^−1^ as those values were recently described as being correlated to survival in melanoma and lymphoma (Salven *et al*, 2000; Ugurel *et al*, 2001). In this series, the median survival was 9 months for patients with VEGF levels lower than 462 pg ml^−1^ and 13 months for other patients (*P*=0.9, Figure 3

). We then tested three different threshold values to assess the impact of the theoretical VEGF load of platelets on survival. None of them made it possible to discriminate a group of good or bad prognosis patients. The same negative results were obtained when different threshold values were tested with plasma VEGF levels: lowest quartile (92 pg ml^−1^, *P*=0.8), median (177 pg ml^−1^, *P*=0.9) and highest quartile (375 pg ml^−1^, *P*=0.63).

### Multivariate analysis

A multivariate analysis of prognostic factors using the Cox model was performed on this series of 87 patients with metastatic breast cancer. The variables introduced in the Cox regression model were the prognostic parameters previously described as being relevant in metastatic breast cancer. These included performance status (0–1 *vs* 2–3), liver involvement (present *vs* absent), lung involvement (present *vs* absent), adjuvant chemotherapy (received *vs* not received), LDH level (⩽normal *vs* >normal), oestrogen receptor status (present *vs* absent) and disease-free interval (⩽24 months *vs* >24 months) (Honig, 1996; Yamamoto *et al*, 1998). Using a model in which all these factors were maintained, IL-6 remained an additional independent prognostic factor for survival. This was confirmed on introducing serum IL-6 levels as a continuous variable (*P*<0.001, hazard ratio [HR]=1.02), or when the different threshold values were tested (Table 2

**Table 2:** Results of the multivariate analysis: significant factors and associated hazard ratio (HR)

**Factor**	***P*-value**	**HR**	**95% confidence interval of HR**
PS>1	<0.001	3.1	1.8–5.6
IL-6>=8 pg ml^−1^	0.007	2.2	1.2–3.8
Prior adjuvant chemotherapy	0.002	2	1.4–4.7

PS=performance status.

): IL-6 ⩾8 pg ml^−1^ (*P*=0.007, HR=2), IL-6 ⩾13 pg ml^−1^ (*P*<0.001, HR=3.2), IL-6 ⩾30 pg ml^−1^ (*P*=0.001, HR=3) and IL-6 ⩾55 pg ml^−1^ (*P*<0.001, HR=5.2).

## DISCUSSION

There is accumulating evidence that the serum levels of angiogenic factors have prognostic significance in human cancers of epithelial or hematological origin (Poon *et al*, 2001). In this study, we have evaluated the clinical significance of serum VEGF and IL-6 levels on 87 metastatic breast cancer patients.

Several studies have reported that metastatic breast cancer patients have elevated serum levels of VEGF as compared with normal controls, as well as with women with primary breast cancer. Three of those studies, performed with the same immuno-assay as the one we used, reported median serum VEGF levels ranging from 186 to 368 pg ml^−1^, with a considerable range between minimum and maximum levels (10 to 2426 pg ml^−1^ in the series of Kraft *et al*, 1999) (Salven *et al*, 1999b; Adams *et al*, 2000). These results are consistent with our findings (median: 302 pg ml^−1^; range 31–3183) confirming the strong correlation between serum VEGF and platelet count (Salgado *et al*, 1999). In this series, the median platelet VEGF concentration, estimated from the ratio between serum VEGF and platelet count, was 1.5 pg 10^−6^ platelets, which is in agreement with results obtained in other series of metastatic cancer patients (Salgado *et al*, 1999; Salven *et al*, 1999a). Furthermore, we have confirmed the correlation between platelet VEGF concentration and IL-6 levels, thus placing further emphasis on the importance of IL-6 on VEGF metabolism (Vermeulen *et al*, 1999; Salgado *et al*, 2002).

As most serum VEGF is released from platelets during blood clotting, plasma VEGF may be a better reflection of ongoing angiogenesis and, accordingly, a better prognostic marker for patients with cancer (Banks *et al*, 1998). To test this hypothesis, we assessed plasma VEGF levels in 73 of our patients for whom an EDTA plasma sample had been collected at the same time as the serum sample. We found that plasma VEGF is strongly correlated to serum VEGF and to platelet count, which is in agreement with previous findings on colorectal cancer patients (George *et al*, 2000; Werther *et al*, 2002). With regard to survival, plasma VEGF levels were not related to prognosis in this subgroup of 73 patients. The strong correlation between serum and plasma VEGF may reflect the fact that plasma VEGF levels are directly related to total blood VEGF levels and correspond to free VEGF after equilibration with platelet levels (George *et al*, 2000). Alternatively, we cannot rule out that the platelet VEGF released following a partial platelet degradation during plasma sampling and storage, may account for part of the EDTA plasma VEGF (Wynendaele *et al*, 1999).

In our series, only 39% patients had detectable levels of IL-6, which is less than previously reported and may be because of the low sensitivity of the ELISA kit we used (detection limit: 8 pg ml^−1^) (Salgado *et al*, 1999; Zhang and Adachi, 1999). We could not find any correlation between serum IL-6 and platelet numbers nor between serum IL-6 and VEGF levels, and this result was maintained when the analysis was restricted to patients who had not received any chemotherapy for their metastatic disease.

With regard to survival, the present results show that high serum IL-6 levels are associated with a poor overall survival, while serum and plasma VEGF levels do not significantly correlate to the outcome. Results of the multivariate analysis show that IL-6 levels remain independent variables for survival when assessed with clinical prognostic markers.

No studies to date have shown any correlation between the survival of metastatic breast cancer patients and serum or plasma levels of VEGF, which is in contrast with several other publications reporting a strong correlation between VEGF expression in the tumour and prognosis in early-stage breast cancers (Gasparini, 2000; Manders *et al*, 2002). Different factors might account for the lack of prognostic value of VEGF in our series. Breast cancer is a heterogeneous disease with variable biological and clinical behaviours, in which clinical evolution is much less predictable than in lymphoma or metastatic renal cell carcinoma. Serum VEGF may reflect the disease status at a given time (i.e. stable or progressive) and fail to mirror important biological properties, such as sensitivity to chemotherapy or to hormone manipulation, that will ultimately be of utmost importance for prognosis (Honig, 1996). This hypothesis is supported by the work of Dirix *et al* (1997), who have reported that the positive association of a short tumour volume-doubling time with elevated bFGF and VEGF serum levels in advanced cancer patients is largely independent from the metastatic pattern and the extent of the disease. An alternative explanation may be the relative importance of VEGF, as compared to other angiogenic factors, in metastatic breast cancer. In a model of human breast carcinoma xenografted into nude mice, VEGF was shown to be critical for tumorigenesis, but not for growth, after the tumours had reached a certain size (Yoshiji *et al*, 1997). The authors hypothesised that VEGF downregulation might be balanced by bFGF, TGF*α* or other angiogenic factors. In metastatic breast cancer, angiogenesis may thus depend on numerous redundant angiogenic factors whose relative importance varies from one patient to another and/or during the evolution of the disease.

Since IL-6 induces VEGF transcription and augments both platelet production and platelet storage of VEGF, it was formerly hypothesised that IL-6 could be regarded as an indirect angiogenic factor acting mainly on VEGF metabolism (Salgado *et al*, 1999; Vermeulen *et al*, 1999). Results presented here do not support this hypothesis. We have confirmed the relation between platelet VEGF content and IL-6, but we have failed to show any association between survival and VEGF, or between survival and platelet VEGF content. On the other hand, the serum level of IL-6 is an independent prognosis factor, which confirms results obtained by Zhang *et al* on a smaller series (Zhang and Adachi, 1999). This suggests that, apart from the regulatory effects of IL-6 on VEGF production, other mechanisms are responsible for the deleterious effects of high serum IL-6 levels on tumour growth, thus explaining their strong correlation with prognosis in metastatic breast cancer patients. Although IL-6 expression in early-stage breast carcinomas has been correlated with low grade, oestrogen receptor status and good prognosis (Fontanini *et al*, 1999; Karczewska *et al*, 2000), several studies have shown that IL-6 may contribute to disease progression, particularly in advanced breast cancer patients. Indeed, IL-6 can modulate steroid hormone responsiveness by increasing aromatase activity and oestradiol 17 beta-hydroxysteroid dehydrogenase activity in malignant tissue and by activating oestrogen receptor-*α* (Duncan *et al*, 1994; Singh *et al*, 1999; Speirs *et al*, 2000). IL-6 may promote cell migration by activating the mitogen-dependent protein kinase pathway, and increase chemotherapeutic resistance by inhibiting the activation of proteases involved in apoptosis (Lotem and Sachs, 1997; Badache and Hynes, 2001). Finally, IL-6 promotes osteoclast formation and inhibits dendritic cell differentiation, thus facilitating metastatic growth (Menetrier-Caux *et al*, 1998; Grano *et al*, 2000). As tumours evolve toward a metastatic phenotype and interfere with other endogenous or exogenous factors, IL-6 activity on cancer cells and their environment might actually shift from growth inhibition and differentiation to proliferation and antiapoptosis (Lu and Kerbel, 1993; Chiu *et al*, 1996; Badache and Hynes, 2001). This could explain the favourable prognosis associated with the presence of tumour IL-6 in early-stage breast cancer (Karczewska *et al*, 2000) and the poor survival associated with high serum IL-6 levels in this series of metastatic breast cancer patients. It is noteworthy that, as in early-stage breast cancer, IL-6 functions as an inhibitor of cancer cell growth in benign prostate hyperplasia (Degeorges *et al*, 1996), but is correlated with a poor survival in stage-D prostate cancer (Nakashima *et al*, 2000).

Finally, as no serum acute-phase protein levels were assessed in this study, we cannot rule out that IL-6 serum levels in this population may merely be associated with a generalised inflammatory response to metastatic disease. IL-6 has been shown to be involved in the physiopathology of paraneoplastic inflammatory syndromes observed in patients with renal-cell carcinoma; a similar phenomenon might occur in metastatic breast cancer patients (Blay *et al*, 1997).

In conclusion, this study shows that high serum IL-6 levels are independently correlated to survival in a relevant population of metastatic breast cancer patients. Our results suggest that IL-6 plays an important role in metastatic breast carcinoma progression *in vivo*. If confirmed on a larger, prospective study, this might prove helpful in identifying patients with a particularly poor prognosis, which is of utmost importance when planning therapy.
